# Impact of Exercise in Hypoxia on Inflammatory Cytokines in Adults: A Systematic Review and Meta-analysis

**DOI:** 10.1186/s40798-023-00584-6

**Published:** 2023-06-29

**Authors:** Mousa Khalafi, Mohammad Hossein Sakhaei, Michael E. Symonds, Saeid Reza Noori Mofrad, Yubo Liu, Mallikarjuna Korivi

**Affiliations:** 1 Department of Physical Education and Sport Sciences, Faculty of Humanities, University of Kashan, Kashan, Iran; 2 Department of Exercise Physiology, Faculty of Sport Sciences, University of Guilan, Guilan, Iran; 3 Centre for Perinatal Research, Academic Unit of Population and Lifespan Sciences, School of Medicine, University of Nottingham, Nottingham, NG7 2UH UK; 4 Institute of Human Movement and Sports Engineering, College of Physical Education and Health Sciences, Zhejiang Normal University, Jinhua City, 321004 Zhejiang China

**Keywords:** Exercise, Hypoxia, Inflammatory cytokines, IL-6, TNF-α, IL-10

## Abstract

**Background:**

Both acute exercise and environmental hypoxia may elevate inflammatory cytokines, but the inflammatory response in the hypoxic exercise is remaining unknown.

**Objective:**

We performed this systematic review and meta-analysis to examine the effect of exercise in hypoxia on inflammatory cytokines, including IL-6, TNF-α and IL-10.

**Methods:**

PubMed, Scopus and Web of Science were searched to identify the original articles that compared the effect of exercise in hypoxia with normoxia on IL-6, TNF-α and IL-10 changes, published up to March 2023. Standardized mean differences and 95% confidence intervals (CIs) were calculated using a random effect model to (1) determine the effect of exercise in hypoxia, (2) determine the effect of exercise in normoxia and (3) compare the effect of exercise in hypoxia with normoxia on IL-6, TNF-α and IL-10 responses.

**Results:**

Twenty-three studies involving 243 healthy, trained and athlete subjects with a mean age range from 19.8 to 41.0 years were included in our meta-analysis. On comparing exercise in hypoxia with normoxia, no differences were found in the response of IL-6 [0.17 (95% CI − 0.08 to 0.43), *p* = 0.17] and TNF-α [0.17 (95% CI − 0.10 to 0.46), *p* = 0.21] between the conditions. Exercise in hypoxia significantly increased IL-10 concentration [0.60 (95% CI 0.17 to 1.03), *p* = 0.006] compared with normoxia. In addition, exercise during both hypoxia and normoxia increased IL-6 and IL-10, whereas TNF-α was increased only in hypoxic exercise condition.

**Conclusion:**

Overall, exercise in both hypoxia and normoxia increased inflammatory cytokines; however, hypoxic exercise may lead to a greater inflammatory response in adults.

**Supplementary Information:**

The online version contains supplementary material available at 10.1186/s40798-023-00584-6.

## Key Points

- Exercise during hypoxia or normoxia can increase IL-6 and IL-10 in healthy adults.
- Only hypoxic exercise increases TNF-α concentration.
- Hypoxic exercise induces a greater increase of IL-10 than normoxic exercise, which is clinically beneficial.
- Hypoxic exercise promotes an inflammatory response, but pro- and anti-inflammatory status remain unchanged.

## Introduction

Exercise training under hypoxic conditions has become an important and popular approach to achieve greater adaptations among athletes and untrained adults [1, 2]. Hypoxia is the body’s response to lowered oxygen tension in tissues during high-altitude exposure or pathological conditions [3, 4]. Hypoxic exercise training enhances performance at sea level [5] with an associated improvement in aerobic and anaerobic energy-supply [6], oxygen flux to working muscles [5], oxygen transport and utilization [5, 7] and non-hematological adaptations [8]. In addition, hypoxic exercise training has been proposed as an effective strategy for improving insulin resistance [9], body composition [10] and health related functions [11]. Hypoxic exercise is also associated with superior hormonal and metabolic responses among young healthy adults [12–14].

Both acute and chronic exercise trainings can influence the immune system and inflammatory response in adults. Up on stress or stimuli, cells produce various inflammatory cytokines, including interleukin-6 (IL-6), tumor necrosis factor-α (TNF-α) and interleukin-10 (IL-10) that are involved in damage and repair mechanisms [15]. The pro- and anti-inflammatory action of cytokines typically relies on the physiological context of the body [16, 17]. For instance, TNF-α primarily acts as pro-inflammatory cytokines, whereas IL-10 acts as important anti-inflammatory cytokine with exercise [16, 17]. IL-6 possesses a dual role and is not only pro-inflammatory, but is also anti-inflammatory and can promote tissue regeneration [16, 17]. In some metabolic conditions, such as those associated with obesity, increased circulating IL-6 concentrations contribute to the development of chronic inflammation [16, 17]. However, during exercise, IL-6 released from skeletal muscle suppresses the rise in TNF-α, upregulates IL-10 production [18, 19], and thereby acts as an anti-inflammatory cytokine [20].

Regardless of exercise type, regular exercise has anti-inflammatory effects in healthy individuals and also in patients with metabolic disorders [21–23]. Exercise increases the production and circulation of inflammatory cytokines that can be dependent on exercise intensity [24–26], and exposure to hypoxia might have the potential to further stimulate the immune response and inflammation. Hypoxia can promote the production of reactive oxygen species (ROS), accumulation of which impairs antioxidant defense system, leading to pro-inflammatory responses by the activation of nuclear factor kappa B (NF-κB) signaling [27–29]. In addition, hypoxic exercise may trigger intracellular energetic sensors, to further stimulate cytokine secretion [30, 31]. In this context, several studies have investigated the effect of exercise during hypoxia and normoxia on inflammatory cytokines with mixed results. To the best of our knowledge, no meta-analysis has investigated the effect of exercise on inflammatory cytokine response under hypoxia in adults. Therefore, our study aimed to clarify the influence of exercise during hypoxia on the changes of inflammatory cytokines, including IL-6, TNF-α and IL-10.

## Methods

Our meta-analysis was performed based on the Preferred Reporting Items for Systematic Reviews and Meta-Analysis (PRISMA) guideline and Cochrane Handbook of Systematic Reviews of Interventions. The protocol was registered prospectively with ID: CRD42022304272.

### Search Strategy

Three major research databases of PubMed, Web of Science and Scopus were searched independently by two reviewers (M Kh and M H S) to identify original articles, published through November 2021. The search was then repeated again in March 2023 to find any new relevant articles. The following key words were combined using “AND” and “OR”: ("exercise training", "exercise", "aerobic training", "resistance training", "physical activity"), ("inflammation", "inflammatory", "cytokine", "adipokine", "interleukin-6", "interleukin6", "IL-6", "IL6", "interleukin-10", "interleukin10", "IL-10", "IL10", "tumor necrosis factor alpha", "tumor necrosis factor-alpha", "TNF-α", "TNFα", and ("hypoxia", "hypobaric", "altitude", "hypox*"). When available in the databases appropriate limits/filers of English language, source type, document type and human were applied. In addition, reference lists of retrieved articles were manually searched with all search terms listed in Additional file 1: Table S1.

### Inclusion and Exclusion Criteria

Randomized and non-randomized crossover or parallel group design trials that compared the exercise in hypoxia with normoxia were included. Further inclusion criteria were as follows: (1) English language, peer-reviewed articles, (2) studies of humans subjects without chronic diseases, (3) studies involving adults with a mean age ≥ 18 years, (4) studies reported the results of one or more inflammatory cytokines, including IL-6, TNF-α and IL-10. Non-original studies such as meta-analyses, animal studies and those investigating the effect of chronic exercise, or involving subjects with chronic disease were excluded.

### Data Extraction and Synthesis

Data from each study were extracted independently by two reviewers (M Kh and M H S), and any disagreements were resolved through discussion or with the assistance of another reviewers (M E S and SR N M). The following data were extracted: (1) study characteristics including study design, (2) participant characteristics including age, health and training status, (3) exercise characteristics including mode, type, intensity, and duration, and (4) hypoxia conditions. Moreover, the data for all inflammatory cytokines (IL-6, TNF-α, IL-10) and their respective assessment protocols (ELISA or others) were also extracted. The mean and standard deviation (SD) values of outcomes at pre- and post-exercise were required to perform the meta-analysis. However, if means and SDs were not reported, these data were extracted from medians, ranges and interquartile ranges [32–34]. In addition, when required, SDs were calculated from standard error (SE) using the formula: $\mathrm{SD}=\mathrm{SE}\times \surd N$. Graph digitizer software was used for extraction of data from published figures. For comparing the exercise in hypoxia with normoxia, mean changes for both conditions were calculated by subtracting pre-exercise from post-exercise values. In addition, SD changes were calculated using a formula recommended in the Cochrane handbook in which conservative values of 0.5 for correlation were assumed. Then, mean changes and their SDs were included, where a positive effect size represented a better outcome with hypoxic exercise. If a study had more than one exercise arm and/or hypoxic condition, all were included. For studies with healthy and unhealthy subjects, only healthy subjects were included. For studies with multi-time point data, we only used the time point closest to the end of exercise. To obtain missing or additional data, the corresponding author was contacted.

### Quality Assessment and Sensitivity Analysis

#### Quality Assessment

The quality of each study was assessed using the PEDro tool independently by two reviewers (M H S and SR N M), and any disagreements were resolved with the assistance of another review authors (M Kh). The PEDro tool consisted of 11 search items listed in Additional file 1: Table S2 [35].

#### Sensitivity Analysis

Sensitivity analysis was performed by omitting each individual study to ensure that the results were not affected by any one of the studies included.

### Statistical Analyses

Meta-analyses were performed using the Comprehensive Meta-Analysis Software, version 2 (CMA2) (Biostat Inc., NJ 07631 USA). Standardized mean differences (CMA) and 95% confidence intervals (CIs) were calculated using the random effect model to determine effect size and generate forest plots. The analyses were conducted in three steps based on the intervention arms, as follows: (1) effect of exercise in hypoxia using pre- and post-intervention means and their SDs, (2) effect of exercise in normoxia using pre- and post-intervention means and their SDs and (3) comparing effect of exercise in hypoxia versus normoxia using mean changes (post-values–pre-values) and their SDs. Separate analyses were conducted for all outcomes, including IL-6, TNF-α and IL-10. In addition, subgroup analyses were conducted based on the exercise workload (relative workload vs. absolute workload) for the main analyses. Interpretation of effect size was followed according to the Cochrane guidelines with 0.2–0.49 indicating small, 0.5–0.79 indicating medium and > 0.8 indicating a large effect size [34]. Heterogeneity among studies was conducted using *I*^2^ test as < 25% indicating low, 25–50% indicating moderate, 50–75% high and > 75% indicating considerable heterogeneity [34]. Publication bias was assessed using visual interpretation of the funnel plots and Egger’s test in which significance was set at *p* < 0.1 [36]. When publication bias was reported using visual interpretation of the funnel plots, the trim-and-fill method was used to address any potential effect.

## Results

### Study Characteristics

**Fig. 1:**
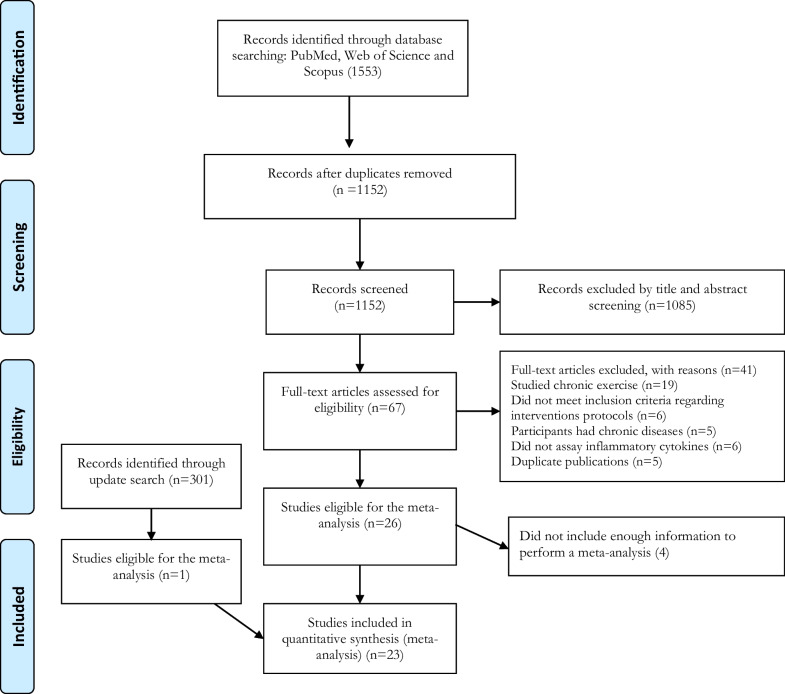
Flow diagram of systematic literature search according to PRISMA

The electronic database search revealed 1553 articles, including 327 from PubMed, 458 from Web of Science and 768 from Scopus, of which 401 articles were excluded based on duplicates screening, and 1085 articles were excluded based on title/abstract screening. Consequently, 67 articles were assessed in full text, of which 45 articles were excluded according to a priori inclusion and exclusion criteria. Through an updated search, one more article met our study criteria [37], and finally, 23 articles were included for the analysis as explained in Fig. 1. Most included studies were within-subject, pre–post-comparisons using a crossover design; two studies [38, 39] were between-subject, pre–post-comparisons using parallel groups. Of these 23 studies, 19 used the widely accepted ELISA kits to assess the inflammatory cytokines. The detailed characteristics of the included studies are presented in Table 1.

**Table 1:** Summary of the demographic characteristics of participants

Study	Participants	Study design	Participant characteristics	Age [years]	Outcomes
Benavente et al. [43]	13 (males)	Within-subject, counterbalanced, Pre–post-comparison	Resistance trained	22.31 ± 2.59	TNF-α, IL-10
Blegen et al. [42]	9 (males)	Within-subject, Pre–post-comparison	Healthy	27.00 ± 4.40	TNF-α
Britto et al. [38]	20 (males)	Between-subject, Pre–post-comparison	Healthy	22.20 ± 1.79	TNF-α, IL-10
Caris et al. [44]	7 (males)	Within-subject, Pre–post-comparison	Healthy	23.00 ± 2.00	IL-6, TNF-α, IL-10
Chen et al. [37]	11 (males)	Within-subject, Pre–post-comparison	Swimmer	21.40 ± 0.99	IL-6
Goods et al. [45]	10 (males)	Within-subject, Pre–post-comparison	Footballers	20.50 ± 1.90	IL-6
Goto et al. [40]	10 (males)	Within-subject, Pre–post-comparison	Endurance athletes	19.80 ± 0.90	IL-6
Goto et al. [46]	10 (males)	Within-subject, Pre–post-comparison	Track-and-field sprinters	20.90 ± 0.30	IL-6
Govus et al. [54]	13 (male = 7, female = 6)	Within-subject, counterbalanced, Pre–post-comparison	Endurance trained and triathlete	28.80 ± 5.30	IL-6
Hagobian et al. [47]	8 (males)	Within-subject, Pre–post-comparison	Trained	24.80 ± 4.00	TNF-α
Hill et al. [55]	10 (male = 9, female = 1)	Within-subject, counterbalanced, Pre–post-comparison	Physically active	21.00 ± 3.16	IL-6, TNF-α, IL-10
Lee et al. [48]	12 (males)	Within-subject, Pre–post-comparison	Healthy	22.00 ± 4.00	IL-6, TNF-α, IL-10
Liara et al. [39]	14 (males)	Between-subject, Pre–post-comparison	Healthy	NOR:25.83 ± 2.90 HYP:24.00 ± 2.34	IL-6, TNF-α, IL-10
Lundby 2004 [56]	8 (male = 6, female = 2)	Within-subject, Pre–post-comparison	Trained	25.00 ± 1.00	IL-6
Mazzeo et al. [49]	8 (males)	Within-subject, Pre–post-comparison	Healthy	23.20 ± 5.60	IL-6
Morrison et al. [58]	11 (ND)	Within-subject, Pre–post-comparison	Footballers	24.20 ± 4.70	IL-6, TNF-α, IL-10
Moura et al. [41]	7 (male = 5, female = 2)	Within-subject, Pre–post-comparison	Healthy	41.00 ± 8.00	IL-6
Santos et al. [29]	9 (males)	Within-subject, Pre–post-comparison	Healthy	24.20 ± 2.17	IL-6, TNF-α, IL-10
Santos et al. [50]	8 (males)	Within-subject, Pre–post-comparison	Healthy	24.40 ± 3.80	IL-6, TNF-α, IL-10
Sevendsen et al. [51]	12 (males)	Within-subject, Pre–post-comparison	Endurance athlete	28.00 ± 4.00	IL-6, TNF-α, IL-10
Wahl et al. [52]	7 (males)	Within-subject, Pre–post-comparison	Healthy	22.10 ± 1.90	IL-6
Żebrowska et al. [53]	12 (males)	Within-subject, Pre–post-comparison	Healthy	24.40 ± 4.00	IL-6, TNF-α
Żebrowska et al. [57]	14 (male = 9, female = 5)	Within-subject, Pre–post-comparison	Healthy	27.10 ± 3.90	IL-6, TNF-α

*IL-6* interleukin 6, *TNFα* tumor necrosis factor-α, *IL-10* interleukin 10, *ND* not-described

### Participant and Exercise Characteristics

Of the 243 participants included, their age ranged from 19.8 [40] to 41 [41] years. There were 17 studies recruited only male participants [29, 37–40, 42–53], five studies included both males and females [41, 54–57] and one study did not report the sex clearly [58]. In addition, all participants were healthy, trained individuals, or athletes (Table 1). A majority of studies used aerobic exercise [29, 39, 41, 42, 44, 47–53, 55–57], four studies used high-intensity interval exercise (HIIE) [45, 46, 54, 58], three studies adopted resistance exercise [37, 38, 43] and one study used combination of HIIE and aerobic exercise [40] as summarized in Table 2.

**Table 2:** Summary of interventions included in study

Study	Exercise type	Exercise description	Exercise workload	Normoxia/hypoxia protocol	Measurement times	Hypoxia-exercise temporality	Type of hypoxia	Altitude (m)	Duration of hypoxia
Benavente et al. [43]	Resistance	Flat barbell press, barbell military press, wide grip lateral pulldown, seated cable row, barbell back squat, and machine leg press, 3 sets of 10 repetition maximum	–	Performed exercise session under either normoxia (< 700 m), or 30-min after arrived to hypoxia (2320 m)	Pre, 15-min Post and 30-min Post	Hypoxia/Normoxia both before and during exercise	TH	2320	ND
Blegen et al. [42]	Aerobic	EX1: 60-min at 60%VO_2max_ EX2: 60-min at 40% VO_2max_	Relative	Participants rested for 30-min and performed exercise session under normoxia (20.93%), or rested for 15-min under normoxia (20.93%), then rested for 15-min under hypoxia (14.65%), and performed exercise session under hypoxia (14.65%)	Pre, 1 h Post, 2 h Post and 24 h Post	Hypoxia/Normoxia both before and during exercise	ND	ND	75-min
Britto et al. [38]	Resistance	1-leg knee extension, 8 sets of 8 repetitions at 80% one repetition maximum	–	Performed exercise session under either normoxia, or hypoxia (14%)	Pre, immediately Post and 4 h Post	Hypoxia/Normoxia during exercise	NH	ND	30-min
Caris et al. [44]	Aerobic	60-min at 50% VO_2max_	Relative	Performed exercise session under either normoxia or hypoxia (13.5%)	Pre, immediately Post and 1 h Post	Hypoxia/Normoxia during exercise	NH	4200	60-min
Chen et al. [37]	Resistance	squat, deadlift, and bench press, six sets with 10 reps at 75% of 1RM	–	Performed exercise session under either normoxia, or hypoxia	Pre, immediately Post and 24 h Post, 48 h post	Hypoxia/Normoxia during exercise		ND	60-min
Goods et al. [45]	HIT	Three sets of 9 × 5-s at maximal sprints with 20-s rest between sprints with 3-min recovery at self-selected low-intensity	Absolute	Performed exercise session under either normoxia (20.9%), or hypoxia (14.5%)	Pre, immediately Post and 1 h Post	Hypoxia/Normoxia during exercise	NH	3000	16.5-min
Goto et al. [40]	HIT/Aerobic	Ten sets of 3-min at 95% VO_2max_ with 60-s recovery at 60% VO_2max_ + 30-min at 85% VO_2max_	Relative	Performed exercise session under either normoxia (20.9%), or hypoxia (14.5%)	Pre, immediately Post, 1 h Post and 2 h Post	Hypoxia/Normoxia during exercise	NH	3000	79-min
Goto et al. [46]	HIT	Three sets of 5 × 6-s at maximal sprints with 30-s rest between sprints and 10-min recovery between sets	Absolute	Participants rested for 10-min, warm-up for 10-min, and performed exercise session under either normoxia (20.9%), or hypoxia (14.5%)	Pre, immediately Post, 1 h Post and 3 h Post	Hypoxia/Normoxia during and post-exercise	ND	3000	225-min
Govus et al. [54]	HIT	Five sets of 4-min intervals at 90% *v*VO_2max_ with 90-s passive recovery	Relative	Performed exercise session under either normoxia (20.9%), or hypoxia (14.5%)	Pre and immediately Post	Hypoxia/Normoxia during exercise	ND	3000	26-min
Hagobian et al. [47]	Aerobic	Until approximately 1500 kcal at 55% of VO_2max_	Absolute	Performed exercise session under either normoxia, or hypoxia	Pre, immediately Post, 2 h post, 4 h Post and 20 h Post	ND	TH	4300	ND
Hill et al. [55]	Aerobic	60-min at 65% VO_2max_	Relative	Performed exercise session under either normoxia (20.9%), or hypoxia (13.5%)	Pre, immediately Post, 1 h Post and 4 h Post	Hypoxia/Normoxia during exercise	NH	4000	60-min
Lee et al. [48]	Aerobic	90-min at 50% VO_2max_	Absolute	Participants rested for 30-min, and performed exercise session under either normoxia (21%), or hypoxia (14%)	Pre and immediately Post	Hypoxia/Normoxia both before and during exercise	NH	3000	120-min
Liara et al. [39]	Aerobic	60-min at 50% VO_2max_	Absolute	Performed exercise session under either normoxia, or hypoxia (13.5%)	Pre and immediately Post	Hypoxia/Normoxia during exercise	NH	4500	60-min
Lundby [56]	Aerobic	60-min at 50% VO_2max_	Absolute	Performed exercise session under either normoxia, or hypoxia (12.4%)	Pre and immediately Post	Hypoxia/Normoxia during exercise	ND	4100	60-min
Mazzeo et al. [49]	Aerobic	50-min at 50% VO2max	Absolute	Performed exercise session under either normoxia, or hypoxia	Pre, 30-min during exercise and 40-min during exercise	Hypoxia/Normoxia during exercise	TH	4300	50-min
Morrison et al. [58]	HIT	Four sets of 4 × 4-s at maximal sprints with 26-s rest between sprints and 146-s passive recovery between sets	Absolute	Performed exercise session under either normoxia (20.9%), or hypoxia (14.5%)	Pre, immediately Post and 3 h Post	Hypoxia/Normoxia during exercise	NH	3000	ND
Moura et al. [41]	Aerobic	6 min walking tests	Absolute	Performed exercise session under either normoxia (20%), or hypoxia (14%)	Pre and immediately Post	Hypoxia/Normoxia during exercise	ND	3000	6-min
Santos et al. [29]	Aerobic	60-min at 70% ventilatory threshold	Relative	Performed exercise session under either normoxia, or hypoxia (12%)	Pre, immediately Post and 1 h Post	Hypoxia/Normoxia during exercise	NH	4200	60-min
Santos et al. [50]	Aerobic	60-min 50% VO_2max_	Absolute	Performed exercise session under either normoxia, or hypoxia (13.5%)	Pre, immediately Post and 1 h Post	Hypoxia/Normoxia during exercise	ND	4200	60-min
Sevendsen et al. [51]	Aerobic	75-min at 70% altitude specific VO_2max_	Relative	Participants warm-up for 10-min, and performed exercise session under either normoxia (20.9%), or hypoxia	Pre, immediately Post and 2 h Post	Hypoxia/Normoxia both during and after exercise	NH	2000	205-min
Wahl et al. [52]	Aerobic	90-min at lactate of 2 mmol∙L-1	Absolute	Performed exercise session under either normoxia (20.8%), or 2 hypoxia (15.9% and 13.2%)	Pre, immediately Post and 3 h Post	Hypoxia/Normoxia during exercise	NH	2000 And 4000	
Żebrowska et al. [53]	Aerobic	After warm-up increasing 30W every 3-min up to maximal exercise intensity	Absolute	Participants warm-up for 3-min and performed exercise session under either normoxia (20.9%), or hypoxia (15.2%)	Pre and immediately Post	Hypoxia/Normoxia both before and during exercise	NH	2500	ND
Żebrowska et al. [57]	Aerobic	40-min at 65% HRmax	Relative	Performed exercise session under either normoxia (20.9%), or hypoxia (15.1%)	Pre, immediately Post and 24 h Post	Hypoxia/Normoxia during exercise	NH	2500	40-min

VO_2max_ maximal oxygen uptake, HR_max_ maximal heart rate, *ND* not described

### Meta-analysis

#### Effect of Exercise in Hypoxia on Inflammatory Cytokines

##### IL-6

Based on twenty intervention arms, exercise increased IL-6 [1.51 (95% CI 1.07 to 1.95), *p* < 0.001] when compared with pre-exercise, with a large effect size (Additional file 1: Fig. S1). We found significant heterogeneity among studies (*I*^2^ = 77.15, *p* < 0.001). Both visual interpretation of funnel plots and Egger’s test (*p* < 0.001) suggested a possible publication bias. After trim-and-fill correction, four studies required adjustments, and the overall change was 1.24 (95% CI 0.80 to 1.68), confirming SMD was still large.

##### TNF-α

Fifteen intervention arms reported TNF-α data and found that exercise intervention significantly increased TNF-α [0.46 (95% CI 0.23 to 0.70), *p* < 0.001] compared with pre-exercise, with a small effect size (Additional file 1: Fig. S2). There was significant heterogeneity among studies (*I*^2^ = 44.08, *p* = 0.03). Visual interpretations of funnel plots and Egger’s test (*p* = 0.006) both suggested publication bias of the included trials. However, following trim-and-fill correction, one study required adjustments, and the overall change was 0.41 (95% CI 0.16 to 0.67), confirming no change in SMD.

##### IL-10

Results from the ten intervention arms revealed that exercise training significantly increased IL-10 [1.04 (95% CI 0.54 to 1.54), *p* < 0.001] compared with pre-exercise, with a large effect size (Additional file 1: Fig. S3). However, the heterogeneity among the studies (*I*^2^ = 72.95, *p* < 0.001) was significant. Visual interpretation of funnel plots did not show publication bias, while Egger’s test (*p* = 0.001) suggested publication bias.

#### Effect of Exercise in Normoxia on Inflammatory Cytokines

##### IL-6

We then analyzed the effect of exercise on IL-6 response to normoxic conditions. A total of 20 trials reported IL-6 data, and our meta-analysis revealed that exercise intervention increased IL-6 [1.26 (95% CI 0.86 to 1.65), *p* < 0.001] with a large effect size (Additional file 1: Fig. S4). We further noticed a significant heterogeneity among the studies (*I*^2^ = 75.33, *p* < 0.001). Based on visual interpretation of funnel plots and Egger’s test (*p* < 0.001), the studies appeared to exhibit publication bias. Nevertheless, after trim-and-fill correction, six studies required adjustments, and the overall change was 0.84 (95% CI 0.44 to 1.25), confirming SMD was still large.

#### TNF-α

Changes in TNF-α were reported in 15 intervention arms. The results revealed that exercise did not significantly affect TNF-α [0.29 (95% CI − 0.08 to 0.66), *p* = 0.12] (Additional file 1: Fig. S5). There was significant heterogeneity among studies (*I*^2^ = 75.94, *p* < 0.001). The funnel plots did not show publication bias; however, Egger’s test (*p* = 0.008) indicated publication bias.

##### IL-10

According to the ten intervention arms, exercise increased IL-10 [0.48 (95% CI 0.27 to 0.70), *p* < 0.001] when compared with pre-exercise, with a small effect size (Additional file 1: Fig. S6). We found no significant heterogeneity among the studies (*I*^2^ = 2.77, *p* = 0.41). For bias assessment, both visual interpretation of funnel plots and Egger’s test (*p* = 0.32) showed no publication bias.

#### Comparing effect of Exercise in Hypoxia Versus Normoxia on Inflammatory Cytokines

##### IL-6

**Fig. 2:**
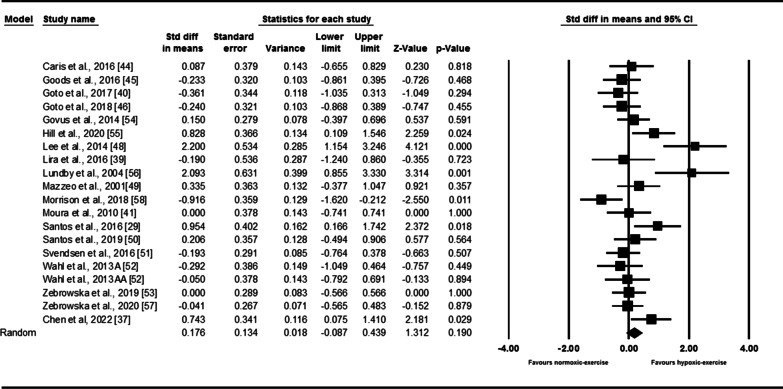
Forest plot of the effects of exercise in hypoxia compared to normoxia on IL-6. Data are reported as SMD (95% confidence limits). SMD: standardized mean difference; Wahl et al. 2013 A [52]: exercise at 2000 m; Wahl et al. 2013 AA [52]: exercise at 4000 m

Next the differential response of IL-6 with exercise was compared between hypoxia and normoxia. From the results of 20 intervention arms, exercise in hypoxia appeared not to affect the IL-6 levels [0.17 (95% CI − 0.08 to 0.43), *p* = 0.17] compared with normoxia (Fig. 2). However, we found a significant heterogeneity among the included studies (*I*^2^ = 64.10, *p* < 0.001). The results from funnel plots and Egger’s test (*p* = 0.01) suggested publication bias. After trim-and-fill correction, two studies required adjustments, and the overall change was 0.27 (95% CI 0.00 to 0.55), confirming SMD slightly increased. Furthermore, subgroup analysis based on exercise workload did not reveal significant changes in IL-6 with both relative (SMD: 0.16, *p* = 0.34) and absolute (SMD: 0.14, *p* = 0.47) workload (Additional file 1: Table S3).

##### TNF-α

**Fig. 3:**
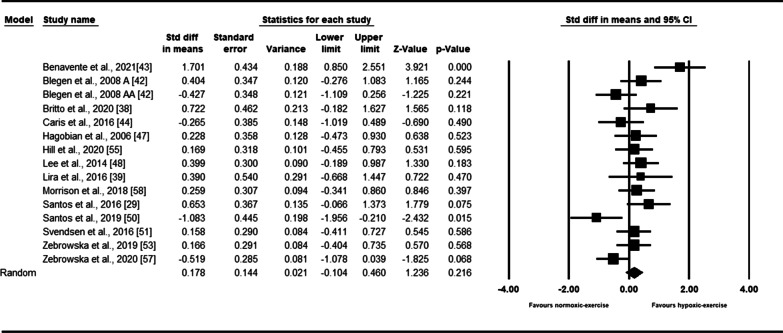
Forest plot of the effects of exercise in hypoxia compared to normoxia on TNF-α. Data are reported as SMD (95% confidence limits). SMD: standardized mean difference. Blegen et al. 2008 A [42]: Low-intensity exercise; Blegen et al. 2008 AA [42]: high-intensity exercise

Based on fifteen intervention arms, exercise in hypoxia did not significantly affect the TNF-α [0.17 (95% CI − 0.10 to 0.46), *p* = 0.21] (Fig. 3). The reported heterogeneity among the studies appeared to be significant (*I*^2^ = 59.88, *p* = 0.002). Visual interpretation of funnel plots suggested publication bias that was not detected by the Egger’s test (*p* = 0.44). However, following trim-and-fill correction, three studies required adjustments, and the overall change was 0.00 (95% CI − 0.30 to 0.31), confirming SMD did not change. In addition, workload subgroup analysis results showed no significant changes in TNF-α with both relative (SMD: 0.13, *p* = 0.55) and absolute (SMD: 0.06, *p* = 0.87) workload (Additional file 1: Table S3).

##### IL-10

**Fig. 4:**
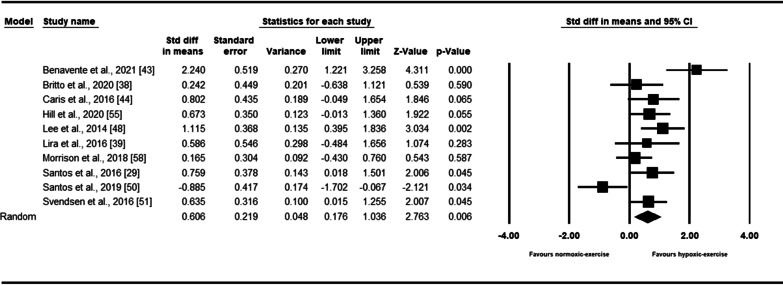
Forest plot of the effects of exercise in hypoxia compared to normoxia on IL-10. Data are reported as SMD (95% confidence limits). SMD: standardized mean difference

Ten intervention arms reported dynamics of IL-10 in response to exercise. The results indicated that exercise in hypoxia increased IL-10 [0.60 (95% CI 0.17 to 1.03), *p* = 0.006] with a moderate effect size (Fig. 4). There was a significant heterogeneity among the included studies (*I*^2^ = 67.48, *p* = 0.001). Funnel plots suggested publication bias, but such bias was not detected by the Egger’s test (*p* = 0.49). After trim-and-fill correction, three studies required adjustments, and the overall change was 0.31 (95% CI − 0.13 to 0.77), confirming SMD slightly decreased. Subgroup analysis results based on exercise workload revealed a significant increase in IL-10 with relative workload (SMD: 0.70, *p* = 0.001), but not with absolute workload (SMD: 0.24, *p* = 0.56) (Additional file 1: Table S3).

### Quality Assessment and Sensitivity Analysis

The quality assessment of all included studies is summarized in Additional file 1: Table S2 which shows the scores ranged from 4 to 7 out of a maximum of 11. For sensitivity analysis, individual studies were removed manually which did not lead to any significant change in the results, as presented in Additional file 1: Table S4.

## Discussion

To the best of our knowledge, this is the first systematic review and meta-analysis to exclusively investigate the effect of exercise in hypoxia on inflammatory cytokines. We demonstrated that when compared with pre-interventions, exercise during both hypoxia and normoxia conditions can increase IL-6 and IL-10; however, an exercise-induced TNF-α increase was found only in hypoxia. Furthermore, exercise in hypoxia did not further stimulate IL-6 or TNF-α but led to a large increase in IL-10. Thus, as a practical and clinical consequence, exercise in hypoxia may not lead to further pro-inflammatory conditions, and pro- and anti-inflammatory status remained in balance.

A recent systematic review showed that exercise increases IL-6 immediately after exercise, especially after intense exercise bouts [59]. In addition, another systematic review suggested that IL-6 increase in response to moderate to high-intensity aerobic and resistance exercise with duration of 30 to 60 min [26]. Our results confirm that exercise stimulates IL-6 regardless of the environment (hypoxia/normoxia conditions). Although the contribution of each of the mechanisms is not clear, several factors contribute to the exercise-induced increase in IL-6. For example, IL-6 can be an anti- and pro-inflammatory cytokine depending on the milieu in which it is present [17]. Beyond its inflammatory action, IL-6 can act as a hormone regulating glucose metabolism, which may enhance muscle glucose uptake and hepatic glucose release [60, 61]. In response to exercise, contracting skeletal muscle is the main source of IL-6 production, for which a decrease in muscle glycogen and subsequent glycogen-depletion enhances IL-6 transcription and its release [61–63]. In addition, exercise is associated with an increased production of intracellular ROS, which may increase IL-6 concentrations [64, 65]. Furthermore, raised catecholamines during exercise can also promote circulating IL-6 [66, 67]. Therefore, the dynamics of IL-6 could be a metabolic-immune response that is mediated in part by increased ROS and/or catecholamine production, together with changes in calcium homeostasis and decreased glucose availability [64]. Hypoxia is associated with decreased oxygen levels, resulting in increased ROS production, which then lead to oxidative damage and causes severe inflammation [28, 68, 69]. Importantly, although both hypoxia [49, 70] and exercise [26, 59] alone enhance IL-6, our meta-analysis emphasized that exercise in hypoxia does not influence IL-6 compared with exercise in normoxia. Elevated IL-6 occurs in reponse to both mechanical and metabolic stress, although the relative contribution of each differs [71]. This hypothesis is supported by Sumi and colleagues who reported that hypoxic exercise is associated with higher metabolic stress and lower mechanical stress than normoxic exercise which lead to similar changes in IL-6 [71]. In addition, it has been proposed that exercise at the same absolute intensity during hypoxia versus normoxia leads to a greater IL-6 response [56]. However, our subgroup analysis by exercise workload did not confirm this.

A previous systematic review reported an increased TNF-α with intense exercise [72]. Our results suggest that normoxic exercise has no effect on TNF-α, but hypoxic exercise increased TNF-α concentration in adults. This response may be explained by a greater metabolic stress occurred with hypoxia is similar to the stress that occurred with intensive exercise [72]. TNF-α is often considered as a main pro-inflammatory cytokine, which release depends on NF-κB signaling. Under stress, nuclear translocation of NF-κB promotes TNF-α transcription [73]. The activation of NF-κB requires mitochondrial ROS [73] and ROS production triggered by hypoxia [74]. Furthermore, activation of hypoxia-inducible factor-1 (HIF-1) also acts as an essential transcription factor that stimulates TNF-α production and may be enhanced by exercise during hypoxia. In this regard, hypoxic exercise is associated with overexpression of HIF-1 mediated by ROS production [43, 75]. Accordingly, the acute effects of hypoxia may be combined with the effects of exercise and consequently lead to increase the TNF-α production by hypoxic exercise.

IL-10 plays an anti-inflammatory role by inhibiting the production and release of key pro-inflammatory cytokines, including TNF-α [76, 77], and is an important clinical marker for assessing chronic inflammation in patients. Pervious meta-analysis by Cabral‐Santos showed that the exercise-induced increase in IL-10 depends on the duration [78], although exercise intensity could also be an important variable [72]. We found that exercise is effective in increasing IL-10 concentrations in both hypoxic and normoxic conditions. The reasons for increased IL-10 remain elusive, but elevated IL-6 from contracting muscles is one mediator for the exercise-induced increase of IL-10 [19]. As we found both normoxic and hypoxic exercise increased IL-6 levels, it is not surprising that IL-10 was also increased. Nevertheless, a greater increase in post-exercise IL-10 with hypoxia may be an indicative of greater metabolic stress. Owing to its anti-inflammatory properties, increased IL-10 with hypoxic exercise may reflect a counter-balancing mechanism to alleviate the impact of increased pro-inflammatory cytokines such as TNF-α [29]. Furthermore, the pronounced increase in IL-10 concentrations with hypoxic exercise may also have an important clinical impact. Therefore, clinical studies with customized hypoxic exercise could potentiate the anti-inflammatory effects of intervention in clinically inflamed patients or those with chronic inflammatory disorders.

Our study has some limitations that should be considered when interpreting the results. The cytokines data were from only one-time point after exercise, in which most studies reported immediately after exercise; therefore, we could not determine the cytokine response during recovery period. There was a significant heterogeneity among all the included studies for some outcomes. We could not perform subgroup analysis for sex due to lack of sufficient details or number of female participants in the included trials. Therefore, any sex-specific response of inflammatory cytokines to hypoxic exercise remains unclear. The differences in protocols or methodologies used to determine the inflammatory cytokines in the included trials (mostly used ELISA and few used other protocols) may also have influenced our meta-analysis results. Furthermore, disparities in terms of the type of exercise did not allow us to perform subgroup analyses to determine the role of aerobic, resistance or high-intensity interval exercise on inflammatory cytokines response.

## Conclusion

Our meta-analysis showed that both hypoxic and normoxic exercises are associated with increased IL-6 and IL-10, whereas only hypoxic exercise increased TNF-α concentration. Indeed, exercise in hypoxia induced a greater increase in IL-10 than normoxic exercise. These results indicated that hypoxic exercise promotes an inflammatory response, but pro- and anti-inflammatory status remained in balance.

## Supplementary Information

**Additional file 1: Table S1.** Search strategy. **Table S2.** Risk of bias assessment. **Table S3.** Summary of subgroup analyses. **Table S4.** Sensitivity analyses. **Fig. S1.** Forest plot of the effects of exercise in hypoxia on IL-6. Data are reported as SMD. SMD: standardized mean difference. Wahl et al, 2013 A [52]: exercise at 2000 m; Wahl et al, 2013 AA [52]: exercise at 4000 m. **Fig. S2.** Forest plot of the effects of exercise in hypoxia on TNF-α. Data are reported as SMD. SMD: standardized mean difference. Blegen et al, 2008 A [42]: Low-intensity exercise; Blegen et al, 2008 AA [42]: high-intensity exercise. **Fig. S3.** Forest plot of the effects of exercise in hypoxia on IL-10. Data are reported as SMD. SMD: standardized mean difference. **Fig. S4.** Forest plot of the effects of exercise in normoxia on IL-6. Data are reported as SMD. SMD: standardized mean difference. Wahl et al, 2013 A [52]: exercise at 2000 m; Wahl et al, 2013 AA [52]: exercise at 4000 m. **Fig. S5.**. Forest plot of the effects of exercise in normoxia on TNF-α. Data are reported as SMD. SMD: standardized mean difference. Blegen et al, 2008 A [42]: Low-intensity exercise; Blegen et al, 2008 AA [42]: high-intensity exercise. **Fig. S6.**. Forest plot of the effects of exercise in normoxia on IL-10. Data are reported as SMD. SMD: standardized mean difference.

## Data Availability

All data and material reported in this systematic review are from peer-reviewed publications.
