# Supplementary material for: Impact of Exercise in Hypoxia on Inflammatory Cytokines in Adults: A Systematic Review and Meta-analysis
Source: Sports Med Open. 2023 Jun 29;9:50. doi: 10.1186/s40798-023-00584-6 (PMC10310663; doi:10.1186/s40798-023-00584-6)
Supplement: Supplementary file 1 — Additional file 1: Table S1. Search strategy. Table S2. Risk of bias assessment. Table S3. Summary of subgroup analyses. Table S4. Sensitivity analyses. Fig. S1. Forest plot of the effects of exercise in hypoxia on IL-6. Data are reported as SMD. SMD: standardized mean difference. Wahl et al, 2013 A [52]: exercise at 2000 m; Wahl et al, 2013 AA [52]: exercise at 4000 m. Fig. S2. Forest plot of the effects of exercise in hypoxia on TNF-α. Data are reported as SMD. SMD: standardized mean difference. Blegen et al, 2008 A [42]: Low-intensity exercise; Blegen et al, 2008 AA [42]: high-intensity exercise. Fig. S3. Forest plot of the effects of exercise in hypoxia on IL-10. Data are reported as SMD. SMD: standardized mean difference. Fig. S4. Forest plot of the effects of exercise in normoxia on IL-6. Data are reported as SMD. SMD: standardized mean difference. Wahl et al, 2013 A [52]: exercise at 2000 m; Wahl et al, 2013 AA [52]: exercise at 4000 m. Fig. S5.. Forest plot of the effects of exercise in normoxia on TNF-α. Data are reported as SMD. SMD: standardized mean difference. Blegen et al, 2008 A [42]: Low-intensity exercise; Blegen et al, 2008 AA [42]: high-intensity exercise. Fig. S6.. Forest plot of the effects of exercise in normoxia on IL-10. Data are reported as SMD. SMD: standardized mean difference. [file 40798_2023_584_MOESM1_ESM.docx]

**Supplementary Table S1. Search strategy**

| Databases | Search strategy | Limits | Results |
| --- | --- | --- | --- |
| PubMed | (("exercise training"[All Fields] OR "exercise"[All Fields] OR "aerobic training"[All Fields] OR "resistance training"[All Fields] OR "physical activity"[All Fields]) AND ("inflammation"[All Fields] OR "inflammatory"[All Fields] OR "cytokine"[All Fields] OR "adipokine"[All Fields] OR "interleukin-6"[All Fields] OR "interleukin6"[All Fields] OR "IL-6"[All Fields] OR "IL6"[All Fields] OR "tumor necrosis factor alpha"[All Fields] OR "tumor necrosis factor alpha"[All Fields] OR "TNF-alpha"[All Fields] OR "TNFalpha"[All Fields] AND ("hypoxia"[All Fields] OR "hypobaric"[All Fields] OR "altitude"[All Fields] OR "hypox*"[All Fields])) | Humans, English | 327 |
| Scopus | ( TITLE-ABS-KEY ( "exercise training"  OR  "exercise"  OR  "aerobic training"  OR  "resistance training"  OR  "physical activity" )  AND  TITLE-ABS-KEY ( "inflammation"  OR  "inflammatory"  OR  "cytokine"  OR  "adipokine"  OR  "interleukin-6"  OR  "interleukin6"  OR  "IL-6"  OR  "IL6"  OR  "Tumor necrosis factor alpha"  OR  "Tumor necrosis factor-alpha"  OR  "TNF-α"  OR  "TNFα"  AND  TITLE-ABS-KEY ( "hypoxia"  OR  "hypobaric"  OR  "altitude"  OR  "hypox*" ) ) | Human, English, Journal | 768 |
| Web of science | 4. #3 AND #2 AND #1  3.(ts=("hypoxia" or "hypobaric" or "altitude" or "hypox*") )  AND LANGUAGE: (English) AND DOCUMENT TYPES: (Article)  2.(ts=("inflammation" or "inflammatory" or "cytokine" or "adipokine" or "interleukin-6" or "interleukin6" or "IL-6" or "IL6" or "Tumor necrosis factor alpha" or "Tumor necrosis factor-alpha" or "TNF-α" or "TNFα") )  AND  LANGUAGE: (English)  AND DOCUMENT TYPES: (Article)  1.(ts=("exercise training" or "exercise" or "aerobic training" or "resistance training" or "physical activity") ) | Article, English | 458 |

**Supplementary Table S2. Risk of bias assessment**

| **Authors & Year** | **Criteria 1** | **Criteria 2** | **Criteria 3** | **Criteria 4** | **Criteria 5** | **Criteria 6** | **Criteria 7** | **Criteria 8** | **Criteria 9** | **Criteria 10** | **Criteria 11** | **Total** |
| --- | --- | --- | --- | --- | --- | --- | --- | --- | --- | --- | --- | --- |
| Benavente et al. 2021 [43] | x | ✓ | x | ✓ | x | x | x | ✓ | x | ✓ | ✓ | 5 |
| Blegen et al, 2008 [42] | x | ✓ | x | ✓ | x | x | x | ✓ | x | ✓ | ✓ | 5 |
| Britto et al, 2020 [38] | ✓ | ✓ | x | ✓ | x | x | x | ✓ | x | ✓ | ✓ | 6 |
| Caris et al. 2016 [44] | ✓ | ✓ | x | ✓ | x | x | x | ✓ | x | ✓ | ✓ | 6 |
| Chen et al. 2022 [37] | ✓ | ✓ | x | ✓ | x | ✓ | ✓ | ✓ | x | ✓ | ✓ | 8 |
| Goods et al, 2016 [45] | x | ✓ | x | ✓ | ✓ | x | x | ✓ | x | ✓ | ✓ | 6 |
| Goto et al, 2017 [40] | ✓ | ✓ | x | ✓ | ✓ | x | x | ✓ | x | ✓ | ✓ | 7 |
| Goto et al, 2018 [46] | x | ✓ | x | ✓ | ✓ | x | x | ✓ | x | ✓ | ✓ | 6 |
| Govus et al. 2014 [54] | x | ✓ | x | ✓ | x | x | x | ✓ | x | ✓ | ✓ | 5 |
| Hagobian et al. 2006 [47] | x | x | x | ✓ | x | x | x | ✓ | x | ✓ | ✓ | 4 |
| Hill et al. 2020 [55] | x | ✓ | x | ✓ | x | x | x | ✓ | x | ✓ | ✓ | 5 |
| Lee et al. 2014 [48] | x | ✓ | x | ✓ | x | x | x | ✓ | x | ✓ | ✓ | 5 |
| Liara et al. 2017 [39] | x | ✓ | x | ✓ | ✓ | x | x | ✓ | x | ✓ | ✓ | 6 |
| Lundby 2004 [56] | x | x | x | ✓ | x | x | x | ✓ | x | ✓ | ✓ | 4 |
| Mazzeo et al. 2001 [49] | x | x | x | ✓ | x | x | x | ✓ | x | ✓ | ✓ | 4 |
| Morrison et al. 2018 [58] | x | x | x | ✓ | x | x | x | ✓ | x | ✓ | ✓ | 4 |
| Moura et al. 2010 [41] | ✓ | ✓ | x | ✓ | ✓ | x | x | ✓ | x | ✓ | ✓ | 7 |
| Santos et al. 2016 [29] | x | ✓ | x | ✓ | x | x | x | ✓ | x | ✓ | ✓ | 5 |
| Santos et al. 2019 [50] | ✓ | ✓ | x | ✓ | ✓ | x | x | ✓ | x | ✓ | ✓ | 7 |
| Sevendsen et al. 2016 [51] | ✓ | x | x | ✓ | x | x | x | ✓ | x | ✓ | ✓ | 5 |
| Wahl et al. 2013 [52] | x | ✓ | x | ✓ | x | x | x | ✓ | x | ✓ | ✓ | 5 |
| Żebrowska et al 2019 [53] | ✓ | ✓ | x | ✓ | x | x | x | ✓ | x | ✓ | ✓ | 6 |
| Żebrowska et al 2020 [57] | ✓ | ✓ | x | ✓ | x | x | x | ✓ | x | ✓ | ✓ | 6 |

(1) Eligibility Criteria specified, (2) Random allocation of participants, (3) Allocation concealed, (4) Groups similar at baseline, (5) Subjects blinded, (6) Therapists blinded, (7) Assessors blinded, (8) Outcome measures assessed in 85% of participants, (9) Intention to treat analysis, (10) Reporting of between group statistical comparison, (11) Point measures and measures of variability reported for main effects. ‘low (✓), ‘high (x) and unclear (?)

**Supplementary Table S3. Summary of subgroup analyses**

|  | Moderators | N | SMD (95% CI) | | P-value | | P-heterogeneity |
| --- | --- | --- | --- | --- | --- | --- | --- |
| **IL-6** |  | | | | | | |
| Workload | Relative | 12 | 0.16 (-0.17 to 0.49) | 0.34 | | 0.07 | |
|  | Absolut | 7 | 0.14 (-0.26 to 0.54) | 0.47 | | 0.001 | |
| **TNF-α** | | | | | | | |
| Workload | Relative | 7 | 0.13 (-0.30 to 0.57) | 0.55 | | 0.08 | |
|  | Absolut | 6 | 0.06 (-0.71 to 0.84) | 0.87 | | 0.03 | |
| **IL-10** | | | | | | | |
| Workload | Relative | 4 | 0.70 (0.34 to 1.05) | 0.001 | | 0.004 | |
|  | Absolut | 4 | 0.24 (-0.57 to 1.06) | 0.56 | | 0.004 | |

**Supplementary Table S4. Sensitivity analyses**

| Hypoxic exercise | IL-6 | Minimum | 1.43 (0.99 to 1.86) | 0.001 |
| --- | --- | --- | --- | --- |
|  |  | Maximum | 1.65 (1.17 to 2.14) | 0.001 |
|  | TNF-α | Minimum | 0.40 (0.19 to 0.61) | 0.001 |
|  |  | Maximum | 0.50 (0.26 to 0.74) | 0.001 |
|  | IL-10 | Minimum | 0.90 (0.43 to 1.37) | 0.001 |
|  |  | Maximum | 1.17 (0.67 to 1.67) | 0.001 |
| Normoxic exercise | IL-6 | Minimum | 1.24 (0.85 to 1.64) | 0.001 |
|  |  | Maximum | 1.41 (1.00 to 1.82) | 0.001 |
|  | TNF-α | Minimum | 0.17 (-0.155 to 0.51) | 0.29 |
|  |  | Maximum | 0.36 (-0.02 to 0.74) | 0.06 |
|  | IL-10 | Minimum | 0.42 (0.19 to 0.64) | 0.001 |
|  |  | Maximum | 0.56 (0.34 to 0.79) | 0.001 |
| Hypoxic versus normoxic exercise | IL-6 | Minimum | 0.05 (-0.17 to 0.27) | 0.66 |
|  |  | Maximum | 0.19 (-0.06 to 0.45) | 0.13 |
|  | TNF-α | Minimum | 0.08 (-0.14 to 0.32) | 0.46 |
|  |  | Maximum | 0.24 (-0.01 to 0.55) | 0.06 |
|  | IL-10 | Minimum | 0.46 (0.09 to 0.82) | 0.01 |
|  |  | Maximum | 0.74 (0.40 to 1.09) | 0.001 |

**
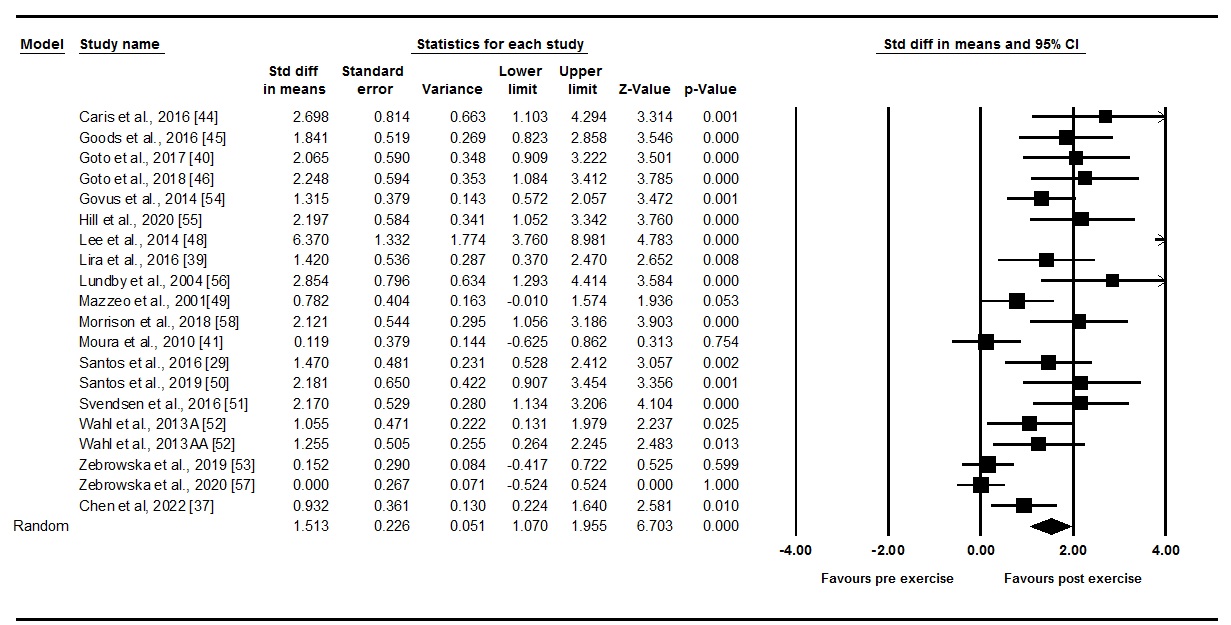
 Supplementary Figure S1.** Forest plot of the effects of exercise in hypoxia on IL-6. Data are reported as SMD (95% confidence limits). SMD: standardized mean difference. Wahl et al, 2013 A [52]: exercise at 2000 m; Wahl et al, 2013 AA [52]: exercise at 4000 m.


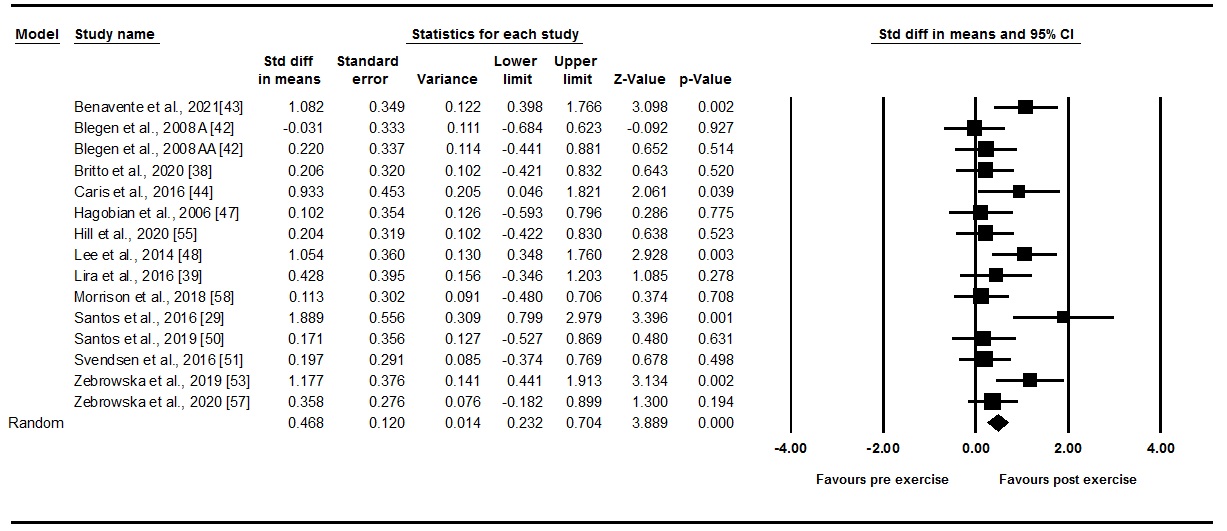


**Supplementary Figure S2.** Forest plot of the effects of exercise in hypoxia on TNF-α. Data are reported as SMD (95% confidence limits). SMD: standardized mean difference. Blegen et al, 2008 A [42]: Low intensity exercise; Blegen et al, 2008 AA [42]: high intensity exercise.


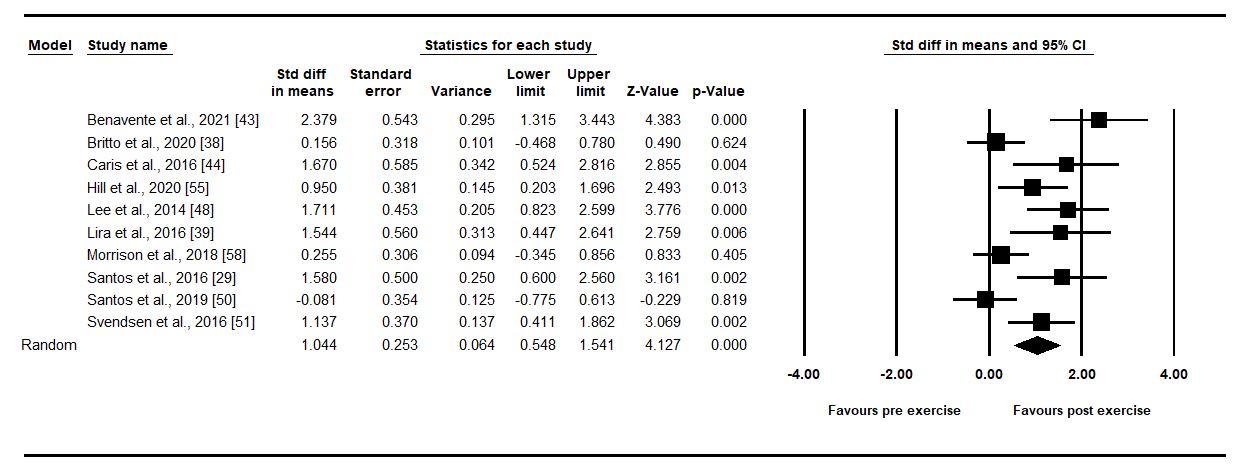


**Supplementary Figure S3.** Forest plot of the effects of exercise in hypoxia on IL-10. Data are reported as SMD (95% confidence limits). SMD: standardized mean difference


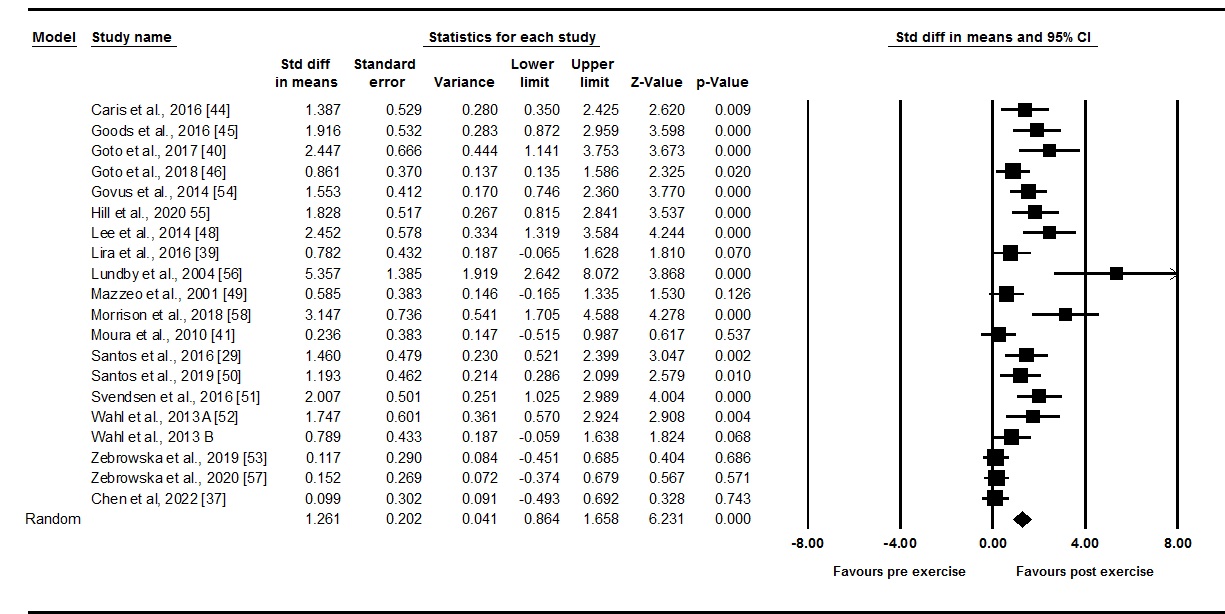


**Supplementary Figure S4.** Forest plot of the effects of exercise in normoxia on IL-6. Data are reported as SMD (95% confidence limits). SMD: standardized mean difference. Wahl et al, 2013 A [52]: exercise at 2000 m; Wahl et al, 2013 AA [52]: exercise at 4000 m.


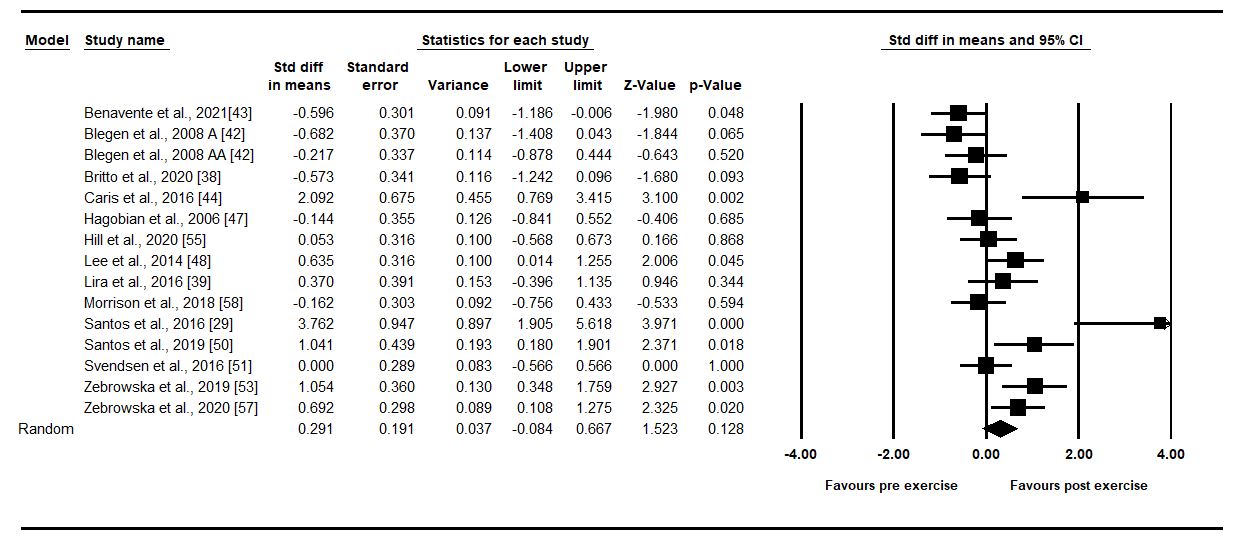


**Supplementary Figure S5.** Forest plot of the effects of exercise in normoxia on TNF-α. Data are reported as SMD (95% confidence limits). SMD: standardized mean difference. Blegen et al, 2008 A [42]: Low intensity exercise; Blegen et al, 2008 AA [42]: high intensity exercise


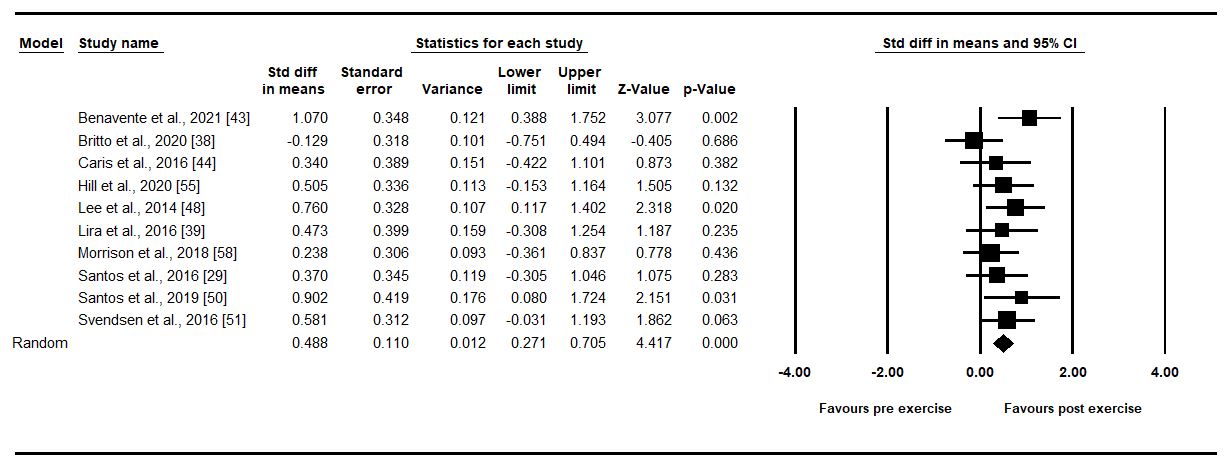


**Supplementary Figure S6.** Forest plot of the effects of exercise in normoxia on IL-10. Data are reported as SMD (95% confidence limits). SMD: standardized mean difference.
